# Mycoremediation of azole antifungal agents using in vitro cultures of *Lentinula edodes*

**DOI:** 10.1007/s13205-019-1733-5

**Published:** 2019-05-09

**Authors:** Agata Kryczyk-Poprawa, Paweł Żmudzki, Anna Maślanka, Joanna Piotrowska, Włodzimierz Opoka, Bożena Muszyńska

**Affiliations:** 1 Department of Inorganic and Analytical Chemistry, Faculty of Pharmacy, Jagiellonian University Medical College, 9 Medyczna Street, 30–688 Kraków, Poland; 2 Department of Medicinal Chemistry, Faculty of Pharmacy, Jagiellonian University Medical College, 9 Medyczna Street, 30–688 Kraków, Poland; 3 Department of Pharmaceutical Botany, Faculty of Pharmacy, Medical College, Jagiellonian University, 9 Medyczna Street, 30–688 Kraków, Poland

**Keywords:** Azole antifungal agents, Bifonazole, Clotrimazole, *Lentinula edodes*, Mycoremediation

## Abstract

Azole antifungal agents are widely used as active ingredients in antifungal pharmaceuticals and agricultural fungicides. An increase in the use of azole antifungals has resulted in an increase in the concentration of these compounds in wastewater and surface water, with potential implications for agriculture. In the present study, bifonazole (BIF) and clotrimazole (CTZ) were selected for investigation because of their widespread use in topical formulations and persistence in the environment. The mycoremediation capacity of BIF and CTZ by mycelia of *Lentinula edodes* in in vitro culture was evaluated. The main aim of this study was to identify the presumable biodegradation products of the investigated active pharmaceutical substances using the LC/MS/MS method. For this purpose, the media were enriched with the following active pharmaceutical ingredients selected for this study: BIF powder, CTZ powder, and BIF cream, each of them at the same concentration of 0.1 mg/mL. Subsequently, thin-layer chromatography coupled with densitometry was used to evaluate the content of BIF and CTZ in mycelium from in vitro cultures of *L. edodes.* The degradation process was found to affect primarily the imidazole moiety of both investigated compounds. In addition, the amounts of undegraded investigated compounds were found to be 4.98, 9.26, and 4.56 mg/g dry weight for BIF powder, CTZ powder, and BIF cream, respectively. Therefore, the findings of this study revealed that *L. edodes* could be considered for remediation of pollution caused by azole antifungal agents.

## Introduction

Civilizational development causes increasing environmental contaminations with heavy metals and chemical substances, e.g., medicines, which pose a threat to the health and safety of humans and animals. Furthermore, the commonly used active pharmaceutical ingredients (APIs) and their degradation products ending up in the environment are currently a global problem. The elimination of pollutants can be achieved, inter alia, by remediation through fungi; this process is known as “mycoremediation”, which is used as a biological tool because of its ability to degrade a wide variety of environmental pollutants, e.g., APIs, and bind with toxic heavy metals present in the environment (Kulshreshtha et al. 2014; Dąbrowska et al. 2018; Kryczyk et al. 2017a). Mycoremediation is a branch of biotechnology that focuses mainly on the assessment of biodegradation capacity of different species of mushrooms and enhancement of their detoxification activity. Because of its desirable characteristics such as high uptake capacity, efficiency, low cost, and safety, mycoremediation is currently becoming an increasingly popular method for eliminating pollutants (Kulshreshtha et al. 2014). However, further efforts are needed to better understand the mechanism underlying the metabolic and enzymatic degradation of the drugs by mushrooms to increase the efficiency of mycoremediation process.

An increase in the consumption of medicines, mainly over-the-counter drugs, is associated with an increase in the risk to the environment. After administration, drugs undergo absorption, metabolism, and excretion into the environment as unchanged drugs and/or their metabolites. Contamination with drugs and their metabolites is also related to their production process and not necessarily to the effective methods of disposal of unused or out-of-date preparations (Larsson 2014; Mudgal et al. 2013). APIs and their metabolites (both biologically active and inactive) may additionally undergo chemical and physical degradation to produce products of unknown structure and activity; thus, pollution due to such agents becomes difficult to control (Muszyńska et al. 2018; Kryczyk et al. 2016).

Antifungal drugs deserve special attention because of their persistence and potential negative impact on the environment (Kahle et al. 2008). Many antifungal drugs are currently used; however, the following groups of drugs play a vital role in the modern therapy of fungal infections: azole derivatives used orally and in topical treatment, squalene epoxidase inhibitors, morpholine derivatives, and polyene antifungal drugs. The presence of antifungal drugs such as ketoconazole, itraconazole, miconazole, clotrimazole (CTZ), and propiconazole in sewage and surface water has been reported earlier by many studies (Lindberg et al. 2010; Peng et al. 2012; Peschka et al. 2007; Van De Steene and Lambert 2008; Van De Steene et al. 2010; Minguez et al. 2016; Huang et al. 2010; Roberts and Thomas 2006). Previous studies have revealed that antifungal drugs present in the natural environment may pose an ecotoxicological threat, especially to aquatic organisms (Casado et al. 2014; Halm-Lemeille and Gomez 2016; Boxall 2004; Yamagishi et al. 2017). Clotrimazole is on the List of Chemicals for Priority Action of OSPAR Commission, because of its persistence in the environment and bioaccumulative and ecotoxicological properties (OSPAR Commission 2005). In the Netherlands, the maximum permissible concentration has been set for CTZ (Mudgal et al. 2013). However, CTZ is not completely removed during sewage treatment, which can cause contamination of agricultural soil through municipal biosolids or wastewater (Sabourin et al. 2011). In addition, it has ecotoxicological effects at environmentally realistic concentrations and affects algal communities at picomolar concentrations (Mudgal et al. 2013; Porsbring et al. 2009). One of the most frequently described methods for effective elimination of contaminations caused by pharmaceutical preparations from the environment is photocatalytic degradation. Antifungal drugs are subject to photocatalytic degradation in the presence of TiO_2_ and ZnO under UVA radiation, but they are resistant to photolysis (Kryczyk et al. 2017b, c; Kryczyk-Poprawa et al. 2018). However, the available literature lacks information on the bioremediation of antifungal drugs. Therefore, the main aim of the present study was to evaluate the effectiveness of the use of edible fungus *Lentinula edodes* (Berk). Pegler in remediating the pollution caused by the azole antifungal agents. *Lentinula edodes* was selected for investigation because of its effectiveness in the mycoremediation of xenobiotics such as cephalosporin antibiotics, synthetic testosterone, and 17α-ethynylestradiol (Muszyńska et al. 2018; Dąbrowska et al. 2018).

Clotrimazole and bifonazole (BIF) were selected for further investigation due to their widespread use in topical preparations and persistence in the environment. In addition, their biodegradation mechanism has not been studied comprehensively, and therefore, no information is available on their role in mycoremediation process. Furthermore, structures of the products formed were identified using the UPLC/MS/MS/technique. Quantitative analysis of selected medicinal substances was carried out by thin-layer chromatography (TLC) coupled with the densitometry method.

## Materials and methods

### Reagents

Bifonazole and CTZ were obtained from Sigma-Aldrich Corp. (St. Louis, MO, USA). HPLC-grade methanol, acetonitrile, and formic acid (98%) were obtained from JT Baker. HPLC-grade dichloromethane was obtained from Merck (Darmstadt, Germany).

Malt extract, glucose, casein hydrolyzate, l-asparagine, adenine, vitamin B_6_, and yeast extract were obtained from Sigma-Aldrich Corp. (St. Louis, MO, USA). Chemicals such as KH_2_PO_4_, MgSO_4_·7H_2_O, FeCl_3_, MnSO_4_·H_2_O, NH_4_Cl, CaCl_2_·6H_2_O, and ZnSO_4_·7H_2_O were obtained from PPH Golpharm (Kraków, Poland). Water (quadruple-distilled) with a conductivity of less than 1 µS cm^−1^ was prepared using S2-97A2 distillation apparatus (ChemLand, Stargard Szczecin, Poland).

## Materials

For the study, commercially available fruiting bodies of *L. edodes* (Berk.) Pegler (mushroom), bought at a supermarket in Poland (2016), were used. The taxonomic identification was performed with MycoKey 4.1 software (http://www.mycokey.com) by an expert Muszyńska. The representative samples of the material used for further studies are stored at the Department of Pharmaceutical Botany, Jagiellonian University Medical College, Kraków, Poland.

### Preparation of *L. edodes* mycelial cultures

The fruiting bodies of *L. edodes* were used to prepare the mycelial culture, within which the obtained mycelium formed the material for further investigation. The fragments of the hymenial part of fruiting bodies were selected to prepare the mycelial culture of *L. edodes*. First, the fragments of the mushroom fruiting bodies were mixed with sterile redistilled water and transferred to the BD Sabouraud Agar with chloramphenicol (under laminar air flow). Then, the cultures were incubated in a thermostat (ST500/B/40 Pol-Eko-Apparatus) at 23 °C ± 2 °C for 2 weeks.

Mycelia derived from the solid medium were used to prepare initial cultures that were cultured on the liquid Oddoux medium. The cultures were shaken at a rate of 140 rpm on a rotary shaker (ALTEL, Poland) and were incubated at a temperature of 23 °C ± 2 °C under 12-h light (900 lx)/12-h dark). The cultures of *L. edodes* were maintained for 2 weeks and later subcultured.

### Experimental mycelial cultures of *L. edodes*

For mycoremediation studies, 25 mg of the investigating compound (BIF or CTZ) was added into 250 mL of sterile liquid medium inoculated with a mycelial culture of *L. edodes.* To perform mycoremediation tests, Canespor^®^ cream (Bayer) containing 10 mg/g BIF, benzyl alcohol, cetostearyl alcohol, cetyl palmitate, 2-octyldodecanol, polysorbate 60, purified water, and sorbitan monostearate were used. First, 2.5 g of Canespor^®^ cream containing 10 mg/g BIF was added to 250 mL of sterile liquid medium inoculated with a mycelial culture of *L. edodes.* Three independent samples were prepared for each investigated compound. Then, the prepared *L. edodes* mycelial culture was cultivated in the same conditions as those for the initial cultures, i.e., cultivated on the modified liquid Oddoux medium. After 14 days of incubation, under these conditions, biomass of *L. edodes* was separated from the medium, mixed with redistilled water, and then freeze-dried using a lyophilizer (FreeZone 4.5, Labconco).

### Sample preparation for analysis

Five grams of the powdered mushroom material (mycelium from in vitro cultures on media containing CTZ and culture media) was extracted with a mixture of methanol and dichloromethane in the ratio of 75:25 (v/v) in an ultrasonic bath at 49 kHz for 30 min (Sonic-2, Polsonic). Merged extracts (300 mL) were concentrated to dryness using a rotary vacuum evaporator at 22 °C ± 2 °C, and the dried extracts were then subjected to UPLC/MS/MS analysis.

### TLC-densitometry method

#### Instruments

The following instruments were used to perform measurements: Densitometer TLC Scanner three controlled by Cats 4 software and equipped with sample applicator Linomat IV (CAMAG, Muttenz, Switzerland); TLC silica gel plates of size 20 × 20 cm (Merck, Germany), which were cut to a size of 10 × 10 cm; a chromatographic chamber of size 18 × 9 × 18 cm (Sigma-Aldrich); an analytical balance (WPA 60/C, Radwag, Poland; with accuracy of 0.1 mg); and a microsyringe of 100 μL volume (Hamilton Comp., USA).

### Chromatographic analysis

The appropriate portions of pharmaceutical standards of BIF and CTZ were dissolved in methanol to obtain standard solutions of the following concentrations: BIF, 0.037 mg/mL and 0.118 mg/mL; CTZ, 0.840 mg/mL and 1.320 mg/mL. To perform the chromatographic separation process of investigated compounds, 5 μl of standard solutions and simulated mixtures was applied to the chromatographic plates (10 × 10 cm) in the form of bands of 8 mm width using the sample applicator Linomat. The chromatograms were developed using a mobile phase composed of *n*-hexane:ethyl acetate:methanol:water:acetic acid (8.4 + 8 + 3 0.4 + 0.2 v/v) to a height of 95 mm, in a chromatographic chamber saturated with the mobile phase for 15 min. Then, chromatograms were dried at room temperature, viewed under the UV lamp at wavelength of *λ* = 254 nm, and then subjected to densitometry analysis. On the basis of the spectra recorded directly from the chromatogram, analytical wavelengths were selected for the quantitative analysis. Peaks of investigated substances in recorded densitograms were found to be well separated; their *R*_F_ values were as follows: ~ 0.62 for BIF and ~ 0.65 for CTZ.

### Validation of the TLC-densitometry method

To validate the applied method, specificity, accuracy, precision, linearity, limit of detection (LOD), and limit of quantification (LOQ) of samples were determined.

Furthermore, accuracy was determined at three concentration levels: 80, 100, and 120%, based on sample analysis of known concentrations and comparison of the results obtained by the validated method with true values, followed by calculation of the recovery percentage.

In addition, the precision of the applied method was determined at three concentrations of substance with respect to reference solutions: 50, 100, and 150%. For each concentration, the experiment was performed in triplicate.

The linearity of substances was also determined by comparing the relationship between peak area (mm^2^) and the amount of test substance applied to the plate (μg/spot). A series of assays were performed for each substance in the following concentration ranges: from 0.037 to 1.406 μg/spot for BIF and from 0.840 to 25.200 μg/spot for CTZ.

The LOD and LOQ were determined from the linearity of each substance in the following concentration ranges: from 0.037 to 0.518 μg/spot for BIF and from 0.840 to 15.200 μg/spot for CTZ, using the following formula: LOD = 3.3 × *S*_*y*_/*a* and LOQ = 10 × *S*_*y*_/*a*, where *S*_*y*_ denotes estimation error and a denotes slope.

### UPLC/MS/MS analysis

The UPLC/MS/MS system consisted of a Waters ACQUITY^®^ UPLC^®^ (Waters Corporation, Milford, MA, USA) coupled to a Waters TQD mass spectrometer (electrospray ionization mode ESI-tandem quadrupole). Chromatographic separation process was carried out using the Acquity UPLC BEH (bridged ethyl hybrid) C_18_ column, 2.1 × 100 mm and 1.7 µm particle size, equipped with Acquity UPLC BEH C18 VanGuard pre-column, 2.1 × 5 mm and 1.7 µm particle size. The column was maintained at 40 °C, and eluted under gradient conditions using from 95 to 0% of eluent A for 10 min, at a flow rate of 0.3 mL min^−1^. Eluent A was composed of water/formic acid (0.1%, v/v), and eluent B was composed of acetonitrile/formic acid (0.1%, v/v). Chromatograms were recorded using Waters eλ PDA detector. Spectra were evaluated in 200–700 nm range with 1.2 nm resolution at a sampling rate of 20 points/s. Mass spectrometry detection settings of Waters TQD mass spectrometer were as follows: source temperature 150 °C, desolvation temperature 350 °C, desolvation gas flow rate 600 L/h, cone gas flow rate 100 L/h, capillary potential 3.00 kV, and cone potential 30 V for the analysis of BIF and 6 V for the analysis of CTZ. Nitrogen was used as both nebulizing and drying gas. The data were recorded in the scan mode ranging from 50 to 1000 *m/z* in time intervals of 0.5 s; eight scans were summed up to obtain the final spectrum. The data acquisition software used was MassLynx V 4.1 (Waters).

### Preparation of samples for UPLC/MS/MS analysis

Approximately 200 μL of the sample, after evaporation, was diluted in 300 μL of the water with the addition of 1% formic acid. The solid-phase extraction cartridges were conditioned with 1 mL of methanol, followed by 1 mL of water. The investigated samples were applied to the cartridges manually and washed with 1 mL of water, followed by the addition of 1 mL of methanol–water (50:50, v/v) mixture, and then eluted with 1 mL of methanol. The prepared samples were injected into the UPLC/MS/MS system for analysis.

### Statistical analysis

The results of remediation tests were statistically analyzed using the Mann–Whitney *U* test that allows the comparison of differences between two independent groups. One-way analysis of variance test with Tukey’s post hoc test of multiple comparisons was also applied to compare the mean of three groups. In both tests, *p* value < 0.05 was considered statistically significant.

## Results and discussion

### Validation of the TLC-densitometry method

The results of validation studies are presented in Table 1. The proposed method was characterized by high sensitivity; LOD for BIF and CTZ was 0.081 and 2.147 μg, respectively. LOQ was estimated to be 0.245 and 6.507 μg, respectively. The percentage recovery of the investigated compounds expressed as mean values for three concentrations was high and ranged from 96.59 to 102.02%. The acceptable precision designated for three concentrations was confirmed by the values of variability coefficients RSD, which were in the range from 0.33 to 2.11%. Linearity was maintained in a wide range: from 0.037 to 1.406 μg/spot for BIF and from 2.520 to 25.200 μg/spot for CTZ. The result of the statistical evaluation of the validated method is presented in Table 2. The linearity was assessed by Mandel’s test (*p* < 0.05 for both analyzed substances), the results of which showed the validity of the selection of quadratic fit. Linear fit was selected for CTZ and square model was selected for BIF. Furthermore, Shapiro–Wilk test confirmed normal distribution of residuals in both cases. The results of Durbin–Watson test indicated significant autocorrelation: positive for BIF and negative for CTZ. The homogeneity of variance of random components in the proposed method was confirmed by Bartlett test (*p *> 0.05).

**Table 1:** Validation of the developed methods with statistical evaluation for TLC-densitometry method

Validation parameters	Bifonazole	Clotrimazole
*R*_F_	~ 0.62	~ 0.65
LOD [μg/spot]	0.081 *a* = 11,405.4 *S*_*y*_ = 280.7	2.147 *a* = 393.5 *S*_*y*_ = 256.2
LOQ [μg/spot]	0.245	6.507
Recovery 80% [%]	$\bar{x}$ = 98.38 *S*_*x*_ = 0.781 RSD = 0.79%	$\bar{x}$ = 101.10 *S*_*x*_ = 0.340 RSD = 0.33%
Recovery 100% [%]	$\bar{x}$ = 102.02 *S*_*x*_ = 0.413 RSD = 0.41%	$\bar{x}$ = 98.33 *S*_*x*_ = 0.831 RSD = 0.84%
Recovery 120% [%]	$\bar{x}$ = 96.59 *S*_*x*_ = 0.468 RSD = 0.49%	$\bar{x}$ = 97.99 *S*_*x*_ = 0.362 RSD = 0.37%
Precision 50% c [mg/mL]	$\bar{x}$ = 0.660 *S*_*x*_ = 0.0063 RSD = 0.95%	$\bar{x}$ = 1.044 *S*_*x*_ = 0.0063 RSD = 0.60%
Precision 100% c [mg/mL^−1^]	$\bar{x}$ = 1.323 *S*_*x*_ = 0.0086 RSD = 0.65%	$\bar{x}$ = 2.100 *S*_*x*_ = 0.0444 RSD = 2.11%
Precision 150% c [mg/mL^−1^]	$\bar{x}$ = 1.983 *S*_*x*_ = 0.0078 RSD = 0.39%	$\bar{x}$ = 3.116 *S*_*x*_ = 0.0204 RSD = 0.66%

*R*_F_ retardation factor, $\bar{x}$ mean value; *S*_*x*_ standard deviation; *RSD* relative standard deviation, *a* the slope of regression line; *S*_*y*_ standard error of the estimate, *m* mass [μg/spot]

**Table 2:** Linear and quadratic equation of examined substances

Substance	Linear equation (*p*)	Quadratic equation (*p*)	*R* ^2^	Mandel^’^s test (*p*)	Shapiro–Wilk test (*p*)	Durbin–Watson test	Bartlett test (*p*)
Bifonazole	*P* = 8615.9·m + 1184.9 (0.000) (0.000)	*P* = − 1547.0·m^2^ +10,841.1·m + 682.2 (0.005) (0.000) (0.007)	0.9913*	10.378 (0.0051)	0.940* (0.906)	0.961*	0.159* (0.991)
Clotrimazole	*P* = 391.1·m + 278.4 (0.000) (0.036)	P = − 0.2510·m^2^ +397.8·m + 252.4 (0.834) (0.000) (0.170)	0.9941	0.045 (0.754)	0.957 (0.603)	0.859	0.043 (0.835)

*P -* peak surface area [mm^2^], *m* - mass [μg/spot], *R*^2^ - coefficient of determination; *(p) - p* significance

*For quadratic equation

### Identification of degradation products of bifonazole and clotrimazole

The identification of the degradation products of BIF and CTZ was performed by UPLC/MS analysis. The proposed structures of the degradation products of BIF and CTZ are shown in Tables 3 and 4, respectively. The degradation process was found to affect primarily the imidazole moiety of both the investigated compounds, leading to its hydroxylation and conjugation with sugars (BP-5) or uronic acids (BP-3, BP-4, BP-6, CP-1, and CP-2). The conjugation of primary metabolites of xenobiotics, e.g., polycyclic aromatic hydrocarbons with sulfate, glucuronic acid, or glucose, was described by previous studies (Cerniglia 1992; Kadri et al. 2017). Furthermore, the degradation involved opening of the imidazole ring (CP-3) and its cleavage (BP-2 and CP-4). Hydroxylation of the imidazole ring, which leads to ring opening and loss of the imidazole moiety, was reported earlier during the photocatalytic degradation process of CTZ (Kryczyk et al. 2017c). The degradation product (2-chlorophenyl) diphenylmethanol (CP-4) was also detected in extracts received after laboratory incubation of agricultural soils (Sabourin et al. 2011). Previous studies have demonstrated that fungi are capable of effectively degrading the phenols due to the activity of their ligninolytic enzymes and indicated the possible degradation process through hydroxylation (Santos and Linardi 2004; Michalowicz and Duda 2007). Due to the oxidative properties of the ligninolytic enzymes produced by *L. edodes*, the degradation of BIF by hydroxylation of the imidazole ring was plausible. CP-4 has been previously classified in European Pharmacopoeia as impurity A ([2-chlorophenyl]diphenylmethanol) (European Pharmacopoeia). Furthermore, the pharmacokinetics studies of CTZ following oral administration showed the presence of (2-chlorophenyl) diphenylmethanol in urine, serum, and bile (Florey 1990). The hydroxylation of phenyl moiety of BIF was also observed during the photocatalytic degradation of this antifungal agent (Kryczyk et al. 2017b). Fragmentation of all the observed degradation products involved cleavage of the imidazole ring, amidine moiety (CP-3), or aminosugar (BP-2), thus yielding carbocations.

**Table 3:**
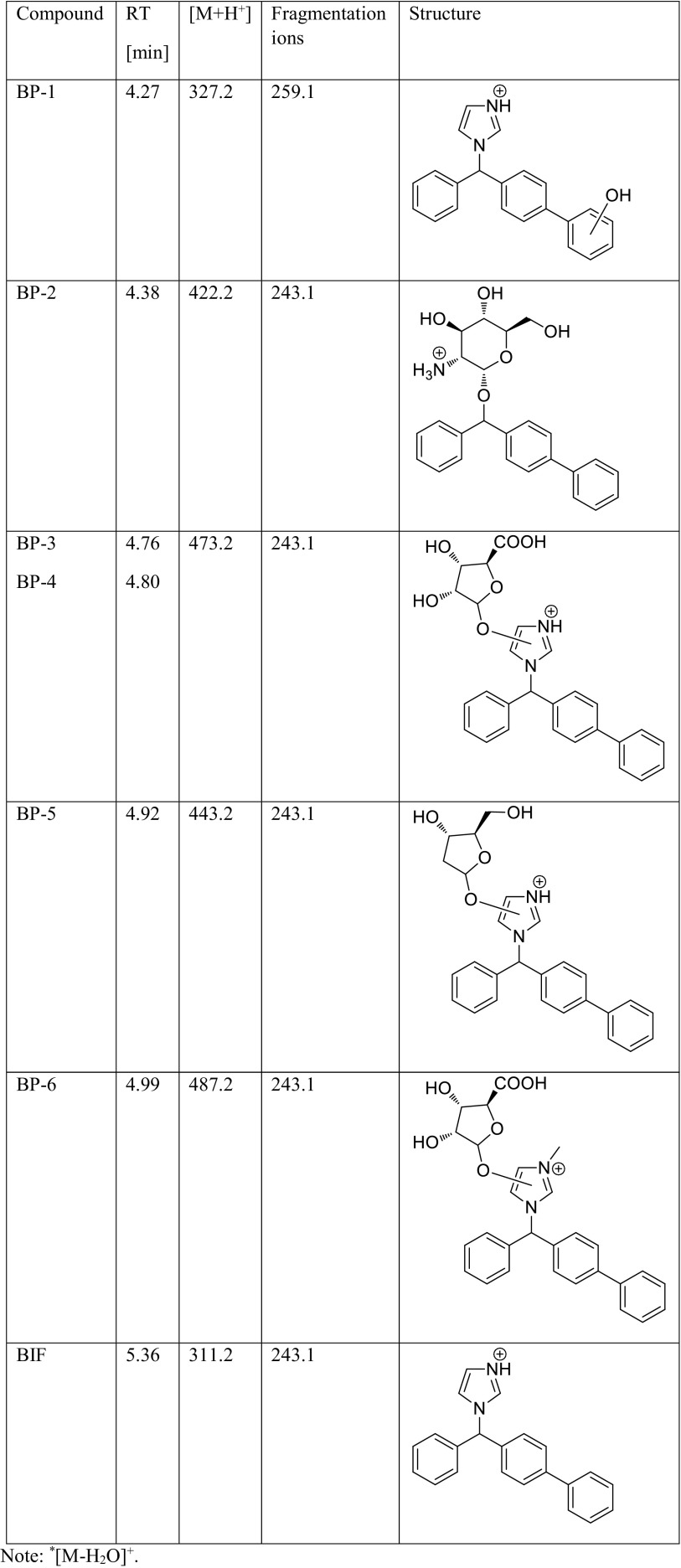
Proposed structures of the biodegradation products of bifonazole

**Table 4:**
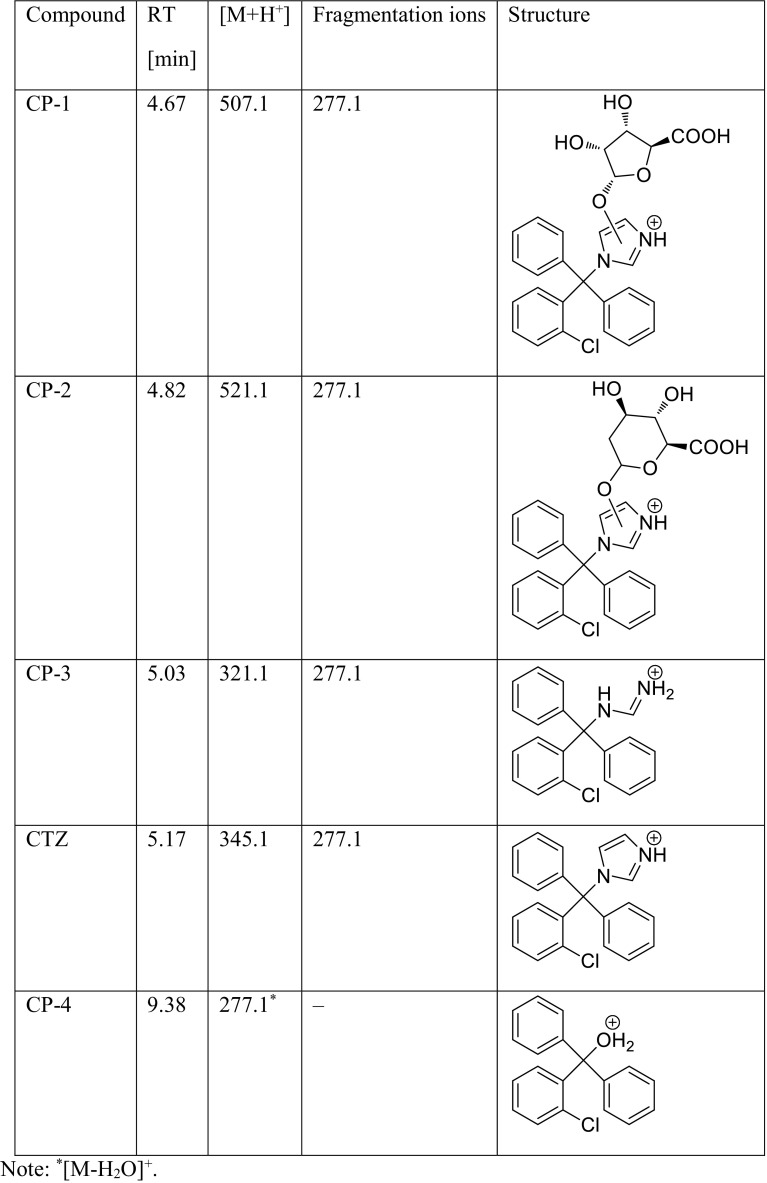
Proposed structures of the biodegradation products of clotrimazole

### Mycoremediation of azole antifungal agents

**Fig. 1:**
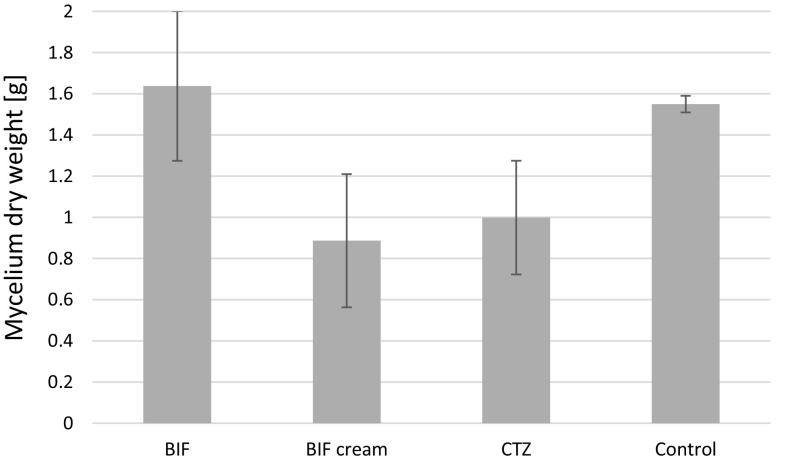
Comparison of dry matter of *Lentinula edodes* mycelium obtained from in vitro cultures, in which 250 mL of culture media was enriched with BIF powder, BIF cream, or CTZ powder

Figure 1 shows a comparison of mycelium growth in in vitro cultures of *L. edodes* with an addition of BIF, BIF cream, and CTZ and without the addition of antifungal drugs (control). The addition of CTZ and BIF in the form of cream caused a slight inhibition of mycelium growth, but the resultant mycelium growth did not differ significantly from that obtained in initial cultures (not enriched with antifungal drugs selected for the study). However, for mycelium enriched with BIF in powder form, most intensive biomass growth was observed.

**Fig. 2:**
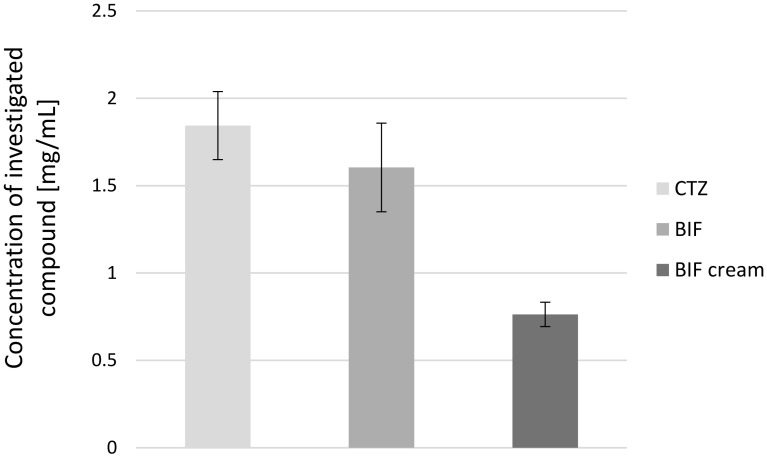
Concentration of BIF and CTZ in mycelium from in vitro culture extract of the *Lentinula edodes* cultured on Oddoux liquid medium enriched with these active substances

The next stage of the study was comparison of the concentrations of the tested substances in *L. edodes* mycelia obtained from in vitro cultures enriched with the investigated antifungal drugs. The concentrations of BIF and CTZ in mycelium from *L. edodes*-extracts on liquid Oddoux medium enriched with these substances in the amount of 25 mg of powder per 250 mL of medium or 2.5 g of BIF cream per 250 mL of medium are shown in Fig. 2. There were no statistically significant differences (*p *< 0.05) between the concentrations of BIF and CTZ in the extract produced from *L. edodes* mycelium.

**Fig. 3:**
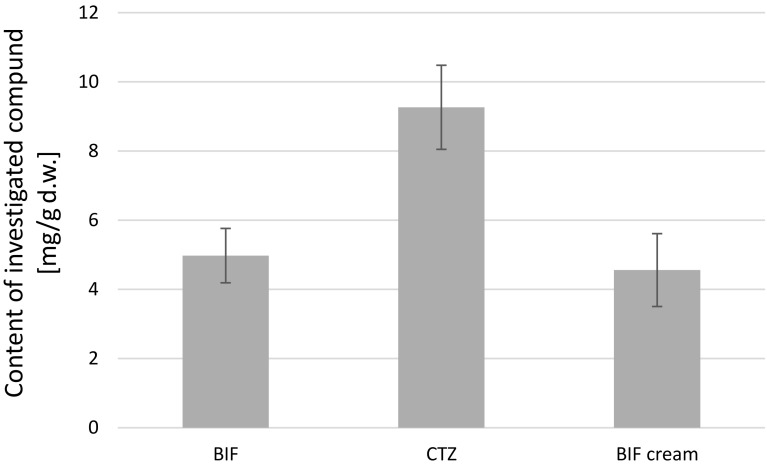
BIF and CTZ content in 1 g of dry matter obtained from in vitro cultures of *Lentinula edodes* cultivated on Oddoux liquid medium enriched with these active substances

The amount of investigated antifungal drugs per gram of mycelium dry matter was also compared with control. The weight of dry mycelium obtained after 4 weeks of incubation was considered for the calculations. The results of analysis of BIF and CTZ content in mycelium obtained from in vitro cultures of *L. edodes* on liquid Oddoux medium enriched with these substances in the amount of 25 mg of powder per 250 mL of medium or 2.5 g of BIF cream per 250 mL of medium are shown in Fig. 3. The total content of the examined substances in the mycelia of *L. edodes* calculated from the determined BIF and CTZ content in 1 g of dry matter obtained from in vitro cultures of the mycelium dry matter was 8.02 and 9.22 mg for BIF and CTZ, respectively, which corresponds to 32.1% and 36.9% of the initial quantity of the investigated compounds BIF and CTZ, respectively.

**Fig. 4:**
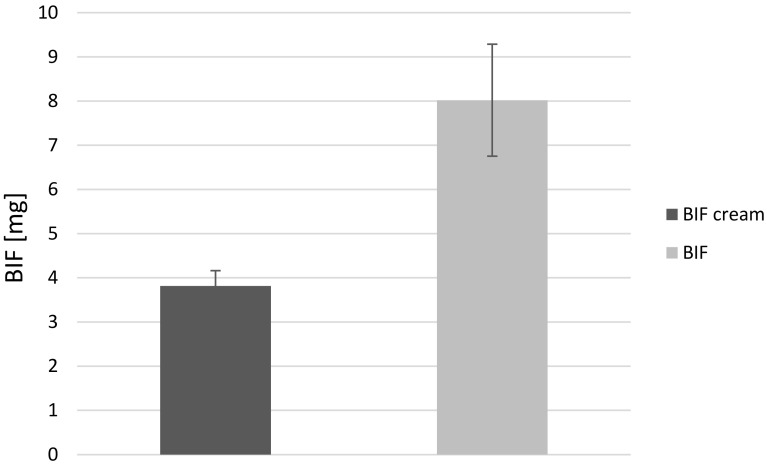
Comparison of the total BIF content of *Lentinula edodes* mycelium obtained from in vitro cultures, in which culture media were enriched with BIF in powder or cream form with a concentration of 0.1 mg mL^−1^ in medium

Figure 4 shows the results of the determination of BIF content in mycelium obtained from in vitro cultures of *L. edodes* on liquid Oddoux medium enriched with this substance in the amount of 25 mg per 250 mL of medium in the form of powder and cream. The use of two different forms of the drug allowed demonstrating their effect on the ability of *L. edodes* to remediate BIF. Statistically significant differences (*p *< 0.05) in the content of BIF between different forms were observed. Bioaccumulation of BIF occurred more efficiently for powder form than for cream form using the same dose of active substance—25 mg per 250 mL of medium. There were no statistically significant differences (*p *< 0.05) between the content of BIF and CTZ in mycelium from in vitro cultures of *L. edodes* when the powder form of both substances was used.

## Conclusion

The main purpose of our study was the assessment of the ability of *L. edodes* to accumulate or degrade azole antifungal agents. The selected drug class is widely used as active ingredients in pharmaceuticals, and previous studies have shown their presence in sewage and surface water. It is worth noting that azole antifungals are used in topical formulations and consequently are released unchanged into the environment. Because of the application of reproducible culture conditions, mycelial in vitro cultures of *L. edodes* allowed for the determination of the degree of bioaccumulation of the examined antifungal drugs. The mean content of investigated compounds in mycelium from in vitro cultures of *L. edodes* was 4.98, 9.26, and 4.56 mg/g dry weight for BIF powder, CTZ powder, and BIF cream, respectively. Bioaccumulation of BIF occurred more efficiently for powder form than for cream form. The mean total amount of BIF accumulated in mycelium from in vitro cultures of *L. edodes* was 9.22 mg and 3.82 mg for BIF powder and BIF cream, respectively. It was shown that the ingredients of the formulation could affect the availability of the compound for mycelium. On the basis of the results obtained, the possibility of the application of examined species in the process of BIF and CTZ remediation was confirmed.
